# Application of Patient-Reported Outcome Measurements in Adult Tumor Clinical Trials in China: Cross-Sectional Study

**DOI:** 10.2196/45719

**Published:** 2024-05-08

**Authors:** Yan Jia, Qi Li, Xiaowen Zhang, Yi Yan, Shiyan Yan, Shunping Li, Wei Li, Xiaowen Wu, Hongguo Rong, Jianping Liu

**Affiliations:** 1 Center for Evidence-Based Chinese Medicine Beijing University of Chinese Medicine Beijing China; 2 Dongzhimen Hospital Beijing University of Chinese Medicine Beijing China; 3 School of Traditional Chinese Medicine Beijing University of Chinese Medicine Beijing China; 4 College of Acupuncture and Massage Beijing University of Chinese Medicine Beijing China; 5 Centre for Health Management and Policy Research Shandong University Shandong China; 6 International Research Center for Medicinal Administration Peking University Beijing China; 7 Peking University Cancer Hospital & Institute Peking University Beijng China; 8 Institute for Excellence in Evidence-Based Chinese Medicine Beijing University of Chinese Medicine Beijing China

**Keywords:** patient-reported outcomes, tumor, cross-sectional study, quality of life, outcome study

## Abstract

**Background:**

International health policies and researchers have emphasized the value of evaluating patient-reported outcomes (PROs) in clinical studies. However, the characteristics of PROs in adult tumor clinical trials in China remain insufficiently elucidated.

**Objective:**

This study aims to assess the application and characteristics of PRO instruments as primary or secondary outcomes in adult randomized clinical trials related to tumors in China.

**Methods:**

This cross-sectional study identified tumor-focused randomized clinical trials conducted in China between January 1, 2010, and June 30, 2022. The ClinicalTrials.gov database and the Chinese Clinical Trial Registry were selected as the databases. Trials were classified into four groups based on the use of PRO instruments: (1) trials listing PRO instruments as primary outcomes, (2) trials listing PRO instruments as secondary outcomes, (3) trials listing PRO instruments as coprimary outcomes, and (4) trials without any mention of PRO instruments. Pertinent data, including study phase, settings, geographic regions, centers, participant demographics (age and sex), funding sources, intervention types, target diseases, and the names of PRO instruments, were extracted from these trials. The target diseases involved in the trials were grouped according to the *American Joint Committee on Cancer Staging Manual, 8th Edition*.

**Results:**

Among the 6445 trials examined, 2390 (37.08%) incorporated PRO instruments as part of their outcomes. Within this subset, 26.82% (641/2390) listed PRO instruments as primary outcomes, 52.72% (1260/2390) as secondary outcomes, and 20.46% (489/2390) as coprimary outcomes. Among the 2,155,306 participants included in these trials, PRO instruments were used to collect data from 613,648 (28.47%) patients as primary or secondary outcomes and from 74,287 (3.45%) patients as coprimary outcomes. The most common conditions explicitly using specified PRO instruments included thorax tumors (217/1280, 16.95%), breast tumors (176/1280, 13.75%), and lower gastrointestinal tract tumors (173/1280, 13.52%). Frequently used PRO instruments included the European Organisation for Research and Treatment of Cancer Quality of Life Core Questionnaire–30, the visual analog scale, the numeric rating scale, the Traditional Chinese Medicine Symptom Scale, and the Pittsburgh Sleep Quality Index.

**Conclusions:**

Over recent years, the incorporation of PROs has demonstrated an upward trajectory in adult randomized clinical trials on tumors in China. Nonetheless, the infrequent measurement of the patient’s voice remains noteworthy. Disease-specific PRO instruments should be more effectively incorporated into various tumor disease categories in clinical trials, and there is room for improvement in the inclusion of PRO instruments as clinical trial end points.

## Introduction

### Background

Patient-reported outcome (PRO) instruments are defined as any report regarding a patient’s health status obtained directly from the patient, excluding interpretation of the patient’s responses by clinicians or other individuals [1]. PRO data consist of information obtained directly from patients concerning their health status, symptoms, treatment adherence, physical and social functioning, health-related quality of life, and satisfaction with health care [2-4]. Serving as noninvasive, comprehensive, and patient-centered metrics, PROs play a pivotal role in enhancing patient engagement, facilitating informed clinical decisions, and improving patient-clinician communication [5-9]. High-quality PRO measures examined in rigorous trials can evaluate treatment effectiveness, assess patient adherence to treatment, guide drug research, and inform health care policies [2,5]. In addition, some PRO instruments could supplement safety data and contribute to the assessment of tolerability (eg, Patient-Reported Outcomes version of the Common Terminology Criteria for Adverse Events [PRO-CTCAE]) [2,5].

In particular, PROs are valuable end points in trials of disabling, chronic, and incurable conditions because they systematically capture the patients’ perspectives in a scientifically rigorous way [3,10,11]. Recognizing their importance, clinical trials focused on tumors are increasingly incorporating PRO instruments as primary or secondary outcomes [12-15]. The European Commission has indicated the priority of preventing cancer and ensuring a high quality of life for patients with cancer within the framework of Europe’s Beating Cancer Plan [16]. The incorporation of PROs in clinical trials offers distinct advantages, including improvements in health-related quality of life, patient-clinician communication, and economic benefits from reduced health care use [17-20]. To uphold best practices in tumor clinical trials that use PROs, several methodological recommendations have emerged in recent years, such as SPIRIT-PRO (Standard Protocol Items: Recommendations for Interventional Trials–Patient-Reported Outcome), CONSORT-PRO (Consolidated Standards of Reporting Trials–Patient-Reported Outcome), SISAQOL (Setting International Standards in Analysing Patient-Reported Outcomes and Quality of Life Endpoints), and other relevant guidelines [2-4,21]. However, PRO measures often receive lower priority in the design of oncology-related clinical trials when compared to survival, imaging, and biomarker-related outcomes [22].

### Objectives

In China, PROs are increasingly being used in clinical trials, but there are challenges as well. A cross-sectional survey of interventional clinical trials conducted in China revealed that only 29.7% of the included trials listed PRO instruments as primary or secondary outcomes [23]. Moreover, there is a notable absence of comprehensive assessments evaluating the application of PRO instruments in tumor clinical trials in China. Unlike previous cross-sectional studies that encompassed all types of clinical trials, our study primarily examined adult tumor clinical trials in China that have listed PRO instruments as primary or secondary outcomes, referencing the methodologies and reporting patterns of a previous study [23]. We extracted the registration information of adult randomized clinical trials conducted in China to systematically analyze the application of PRO instruments in tumor clinical trials, aiming to evaluate the application of PRO instruments in adult tumor clinical trials in China and provide potential directions for further investigation.

## Methods

### Study Design

This cross-sectional study was designed to describe the characteristics of adult tumor clinical trials conducted in China between January 1, 2010, and June 30, 2022, that listed PRO instruments as primary or secondary outcomes. All clinical trials should be registered, and data of clinical trials were collected from 2 clinical trial registries, namely ClinicalTrials.gov and the Chinese Clinical Trial Registry, with public disclosure. We conducted data retrieval and export in July 2022. The clinical trials covered 34 provincial-level administrative regions in accordance with the 2019 version of China’s administrative divisions. We further sought to describe the PRO instruments frequently used in trials encompassing diverse target tumor conditions.

### Data Collection Strategy

**Figure 1:**
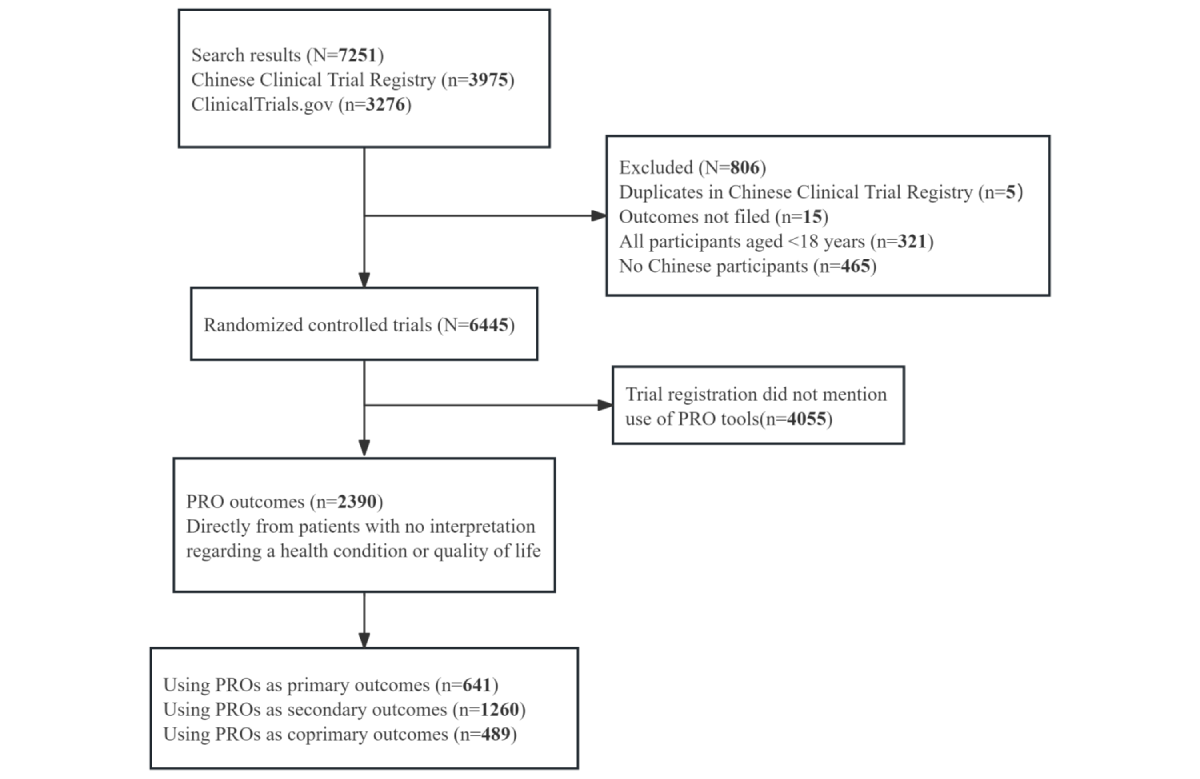
Trial exclusion and classification criteria. PRO: patient-reported outcome.

This study focused on interventional randomized clinical trials conducted in China involving participants aged ≥18 years (Figure 1). Duplicate trials with 2 registration identification numbers were treated as a single trial (ClinicalTrials.gov records were retained). The evaluation of tumor clinical trials included three types of information: (1) basic information (registration number, registration date, scientific name, recruiting country, and other information), (2) key information (outcome, target disease, and age and sex of participants), and (3) characteristic information (main sponsor’s location, study settings, number of setting centers, study stage, funding source, and intervention type).

### Data Classification

PRO instruments were defined by the US Food and Drug Administration in 2009 [1] as any report about a patient’s health status obtained directly from the patient, excluding interpretation of the patient’s response by clinicians or other individuals. Trials using PRO instruments as primary or secondary outcomes were considered PRO trials. On the basis of a previous study of PRO labeling of new US Food and Drug Administration–approved drugs (2016-2020) [24], eligible trials were classified into four groups: (1) trials that listed PRO instruments as primary outcomes, (2) trials that listed PRO instruments as secondary outcomes, (3) trials that listed PRO instruments as coprimary outcomes, and (4) trials without any mention of PRO instruments.

### Statistical Analysis

Data related to the characteristics of the included trials (clinical phase, study setting, participant age and sex, region of the primary sponsor, setting center, number of PROs, funding source, and type of intervention) were extracted independently by 2 authors with a predesigned data extraction table. Owing to the varied categories and wide variation of target diseases, we classified similar target diseases based on classifications from the *American Joint Committee on Cancer Staging Manual, 8th Edition* (Multimedia Appendix 1). On the basis of this categorization of diseases, we consolidated the PRO instruments used in each trial to identify those used most frequently. We conducted quantitative analysis only on items that listed the names of PRO instruments for a more detailed understanding of the commonly used evaluation tools. All data analyses were performed using Stata (version 14.0; StataCorp LLC).

### Ethical Considerations

According to the Common Rule (45 CFR part 46) of the US Department of Health and Human Services (Office for Human Research Protections), this study is exempt from institutional review board approval and the requirement for informed patient consent because it did not involve clinical data or human participants. This study followed the STROBE (Strengthening the Reporting of Observational Studies in Epidemiology) reporting guidelines designed for observational studies in epidemiology.

## Results

### Trial Characteristics

Table 1 presents a comprehensive overview of the included trials. The study included 7251 tumor-focused randomized controlled trials conducted in China between January 1, 2010, and June 30, 2022. Of these 7251 trials, 3276 (45.18%) were sourced from ClinicalTrials.gov, and 3975 (54.82%) were identified from the Chinese Clinical Trial Registry. Of these 7251 trials, after excluding 806 (11.12%) trials (n=5, 0.6% duplicates; n=465, 57.7% non-Chinese trials; n=321, 39.8% trials involving children; and n=15, 1.9% trials with incomplete reports), 6445 (88.88%) eligible trials were identified for analysis.

**Table 1:** Characteristics of all identified trials and patient-reported outcome (PRO)–related trials.

Characteristics		Trials (n=6445), n (%)	PRO trials (n=2390), n (%)
**Phase**			
	Early phase^a^	1317 (20.43)	575 (24.06)
	2	873 (13.55)	218 (9.12)
	3	1004 (15.58)	284 (11.88)
	4	779 (12.09)	269 (11.26)
	Other^b^	1514 (23.49)	537 (22.47)
	Unclear	958 (14.86)	507 (21.21)
**Setting**			
	Hospital	6034 (93.62)	2256 (94.39)
	Community	3 (0.05)	3 (0.13)
	Other^c^	300 (4.65)	96 (4.02)
	Unclear	108 (1.68)	35 (1.46)
**Age (y)**			
	≥18	6098 (94.62)	2252 (94.23)
	>65	100 (1.55)	42 (1.76)
	Unclear	247 (3.83)	96 (4.02)
**Sex**			
	Male only	267 (4.14)	107 (4.48)
	Female only	1000 (15.52)	410 (17.15)
	Male and female	5170 (80.22)	1869 (78.2)
	Unclear	8 (0.12)	4 (0.17)
**Regions of China**			
	Southwest	517 (8.02)	207 (8.66)
	Northeast	185 (2.87)	83 (3.47)
	North	797 (12.37)	321 (13.43)
	Northwest	173 (2.68)	63 (2.64)
	East	3745 (58.11)	1309 (54.77)
	South	682 (10.58)	286 (11.97)
	Central	340 (5.28)	120 (5.02)
	Other^d^	6 (0.09)	1 (0.04)
**Centers involved**			
	Single	5626 (87.29)	2046 (85.61)
	Multiple	716 (11.11)	312 (13.05)
	Unclear	103 (1.6)	32 (1.34)
**PRO instruments used**			
	1-3	N/A^e^	2144 (89.71)
	4-6	N/A	218 (9.12)
	7-9	N/A	25 (1.05)
	≥10	N/A	3 (0.13)
**Funding source**			
	Industry	752 (11.67)	186 (7.78)
	Nonindustry institutions	5443 (84.45)	2120 (88.7)
	Combination^f^	219 (3.4)	71 (2.97)
	Unclear	31 (0.48)	13 (0.54)

^a^The early phase trials included a clinical pretest as well as phase 0 and phase 1 trials.

^b^Diagnostic new technique clinical study, inspection technology, and trials involving multiple phases.

^c^Rehabilitation center, nursing home, campus, centers for disease control, home, and research institute.

^d^The trials were conducted in China, but their sponsor was based overseas.

^e^N/A: not applicable.

^f^Combination trials were funded partly by industry and partly by nonindustry institutions, such as universities, hospitals, and so on.

Of the 2,155,306 participants recruited in all included trials, 139,297 (6.46%) were involved in trials with PRO instruments as primary outcomes, 400,064 (18.56%) in trials with PRO instruments as secondary outcomes, and 74,287 (3.45%) in trials with PRO instruments as coprimary outcomes. Among the 6445 trials included, 2390 (37.08%) used PRO instruments as either primary or secondary outcomes, while 4055 (62.92%) did not use any PRO instrument.

The majority of the studies (6098/6445, 94.62%) did not impose any age restrictions on participants (children were excluded). In trials involving PROs, the proportion of older participants (aged >65 y; 42/2390, 1.78%) was slightly higher than in those without PROs (100/6445, 1.55%). Among all trials that incorporated PRO measurements, 17.15% (410/2390) included only female participants, while 4.48% (107/2390) included only male participants. Furthermore, in trials involving only female participants, the vast majority (974/1000, 97.4%) studied breast and female reproductive organ tumors. In trials exclusively involving male participants, more than half (135/267, 50.5%) centered around male genital organ tumors.

Regarding trial phases, of the 6445 clinical trials, early phase trials were the most prevalent (n=1317, 20.43%), followed by phase 3 trials (n=1004, 15.58%), phase 2 trials (n=873, 13.56%), and phase 4 trials (n=779, 12.09%). Of the 2390 PRO-related trials, early phase trials were again the most common (n=575, 24.06%), followed by phase 3 trials (n=284, 11.88%), phase 4 trials (n=269, 11.26%), and phase 2 trials (n=218, 9.12%).

Most of the trials (6034/6445, 93.62%) were conducted in hospitals, with hardly any (3/6445, 0.05%) conducted in community settings. More than half of the primary sponsors were located in eastern China (3745/6445, 58.11%), followed by northern (797/6445, 12.37%) and southern (682/6445, 10.58%) China, while 18.85% (1215/6445) of the primary sponsors were situated in other regions of China, such as the southwestern, central, northwestern, and northeastern regions. Similar patterns were observed for studies involving PROs. The majority of the major sponsors (1916/2390, 80.17%) originated from the eastern, northern, and southern regions of China, while 19.79% (473/2390) hailed from the southwestern, central, northeastern, and northwestern regions. There were differences in the proportions of PRO trials were noted among different provinces; the distribution of PRO instruments across Chinese provinces can be found in Multimedia Appendix 2.

Moreover, 87.29% (5626/6445) of the trials were single-center trials, and only 11.11% (716/6445) were multicenter trials. Similar phenomena were observed for PRO-related studies, but multicenter trials accounted for a slightly higher percentage (312/2390, 13.05%). Of the 2390 PRO trials, 2144 (89.71%) used 1 to 3 PRO instruments, followed by 4 to 6 (n=218, 9.12%) and 7 to 9 (n=25, 1.05%) PRO instruments. The majority of the trials were nonindustry-funded trials (5443/6445, 84.45%), while 11.67% (752/6445) were industry-funded trials.

Table 2 shows the frequency of intervention types used across different trial classifications. The data indicated that more than a third of the included trials used drugs as the intervention (2496/6445, 38.73%), followed by combination therapies (1350/6445, 20.95%) and surgery (1044/6445, 16.2%). Among clinical trials involving drug interventions, nearly four-tenths (989/2496, 39.62%) used PRO instruments as their outcomes. Trials using drugs as the intervention exhibited a higher incidence of using PRO instruments as their primary or coprimary outcomes (468/989, 47.32%) compared to trials using other intervention types.

**Table 2:** Frequency of intervention types used across different trial classifications.

Intervention	Trials (n=6445), n (%)	PRO^a^ trials			
		Trials, n/N (%)	Primary outcome, n/N (%)	Secondary outcome, n/N (%)	Coprimary outcome, n/N (%)
Drug	2496 (38.73)	989/2496 (39.62)	250/989 (25.28)	521/989 (52.68)	218/989 (22.04)
Biological	400 (6.21)	73/400 (18.25)	8/73 (10.96)	61/73 (83.56)	4/73 (5.48)
Surgery	1044 (16.2)	329/1044 (31.51)	124/329 (37.69)	184/329 (55.93)	21/329 (6.38)
Radiation	187 (2.9)	57/187 (30.48)	11/57 (19.3)	44/57 (77.19)	2/57 (3.51)
Combination	1350 (20.95)	375/1350 (27.78)	52/375 (13.87)	297/375 (79.2)	26/375 (6.93)
Other^b^	968 (15.02)	567/968 (58.57)	196/567 (34.57)	153/567 (26.98)	218/567 (38.45)

^a^PRO: patient-reported outcome.

^b^Other interventions included acupuncture, physical exercise, and psychosocial treatment.

### Conditions and Participants

**Figure 2:**
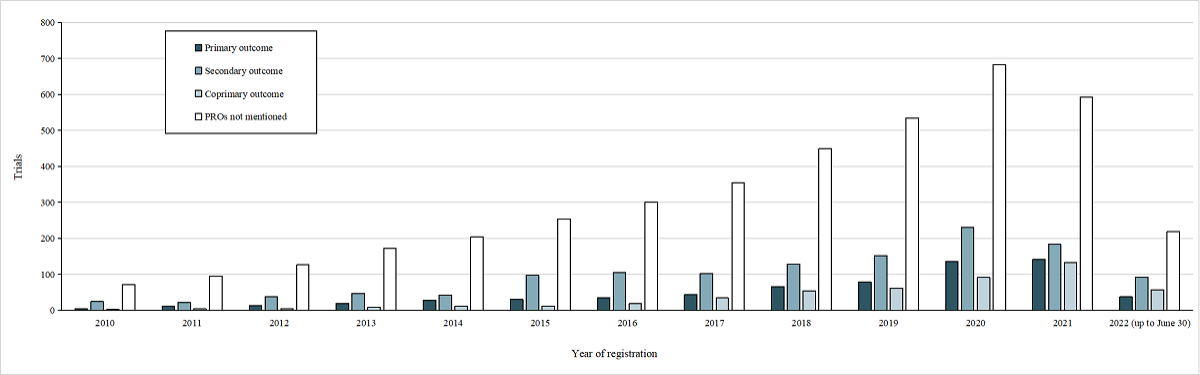
Number of tumor clinical trials analyzed. PRO: patient-reported outcome.

The annual counts of tumor clinical trials are listed in Figure 2. During the study period—from January 1, 2010, to June 30, 2022—the number of tumor clinical trial registrations exhibited a consistent upward trajectory, paralleled by a commensurate increase in the number of clinical trials related to PROs.

**Figure 3:**
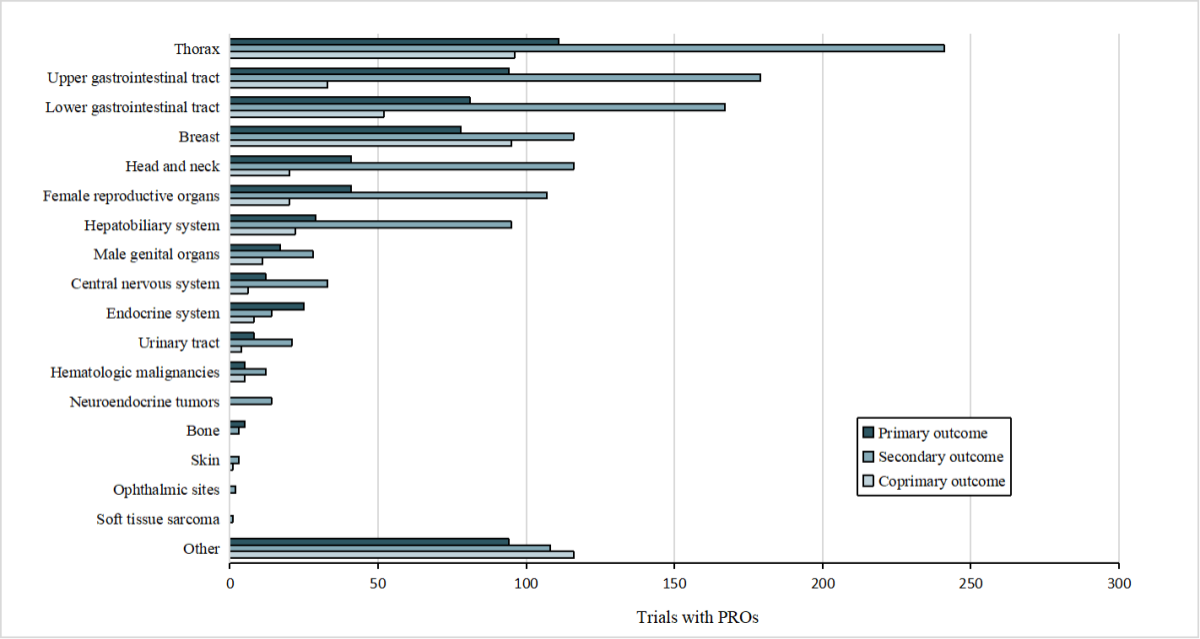
Number of trials with patient-reported outcomes (PROs).

**Figure 4:**
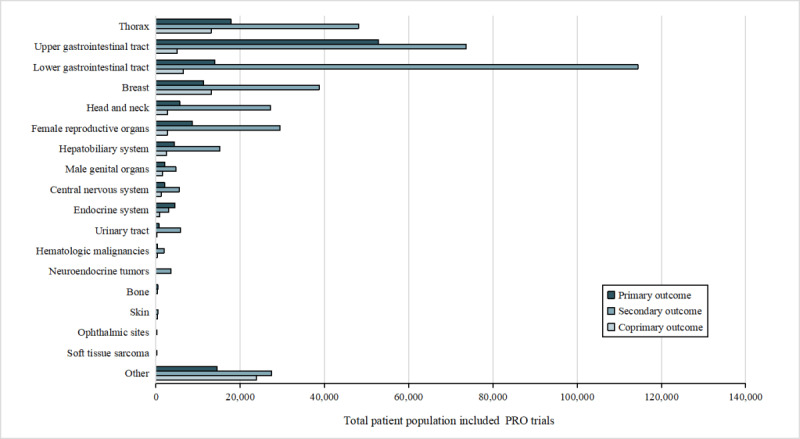
Number of participants with patient-reported outcomes (PROs).

Figures 3 and 4 depict the distribution of trial counts and corresponding participant numbers across different tumor types, respectively, wherein PROs served as outcomes. Among the 2390 tumor-related trials that used PRO instruments as primary or secondary outcomes, the top 5 tumors were thorax (448/2390, 18.74%), upper gastrointestinal tract (306/2390, 12.8%), lower gastrointestinal tract (300/2390, 12.55%), breast (289/2390, 12.09%), and head and neck (177/2390, 7.41%) tumors. Trials regarding female reproductive organ (168/2390, 7.03%) and hepatobiliary system (146/2390, 6.11%) tumors were also frequently observed. Male genital organ tumors (56/2390, 2.34%), central nervous system tumors (51/2390, 2.13%), endocrine system tumors (47/2390, 1.97%), and urinary tract tumors (33/2390, 1.38%) all accounted for proportions ranging from 1% to 5%, and hematologic malignant tumors (22/2390, 0.92%), neuroendocrine tumors (14/2390, 0.59%), bone tumors (8/2390, 0.33%), skin tumors (4/2390, 0.17%), ophthalmic tumors (2/2390, 0.08%), and soft tissue sarcoma (1/2390, 0.04%) constituted <1% of the trials.

Among the 613,648 participants enrolled in these PRO trials, 134,940 (22%) were diagnosed with lower gastrointestinal tract tumors, 131,470 (21.42%) with upper gastrointestinal tract tumors, and 79,068 (12.88%) with thorax tumors. Furthermore, there were a number of patients with breast tumors (63,238/613,648, 10.31%), female reproductive organ tumors (440,975/613,648, 6.68%), head and neck tumors (35,642/613,648, 5.81%), or hepatobiliary system tumors (22,044/613,648, 3.59%), each involving >10,000 patients. By contrast, conditions with <10,000 participants encompassed central nervous system tumors (8897/613,648, 1.45%), endocrine system tumors (8472/613,648, 1.38%), male genital organ tumors (8357/613,648, 1.36%), urinary tract tumors (6784/613,648, 1.11%), neuroendocrine tumors (3539/613,648, 0.58%), hematologic malignant tumors (2629/613,648, 0.43%), bone tumors (825/613,648, 0.13%), skin tumors (311/613,648, 0.05%), ophthalmic tumors (274/613,648, 0.04%), and soft tissue sarcoma (266/613,648, 0.04%).

### PRO Instruments Used in Clinical Trials

Table 3 presents the number of explicitly specified PROs where trials precisely listed the names of the PRO instruments and the number of implicitly specified PROs where trials referenced patients’ subjective feelings without specifying the instruments used, separately for the 3 trial types. Specifically, the trial that specified the PRO instruments used was classified into “explicitly specified PROs,” and the trial that did not specify the instruments used was classified into “implicitly specified PROs.” It was evident that in primary and coprimary outcome trial sets, a greater number of trials explicitly listed the PRO instruments compared to those that did not specify the instruments used. Among the 3 trial types, the coprimary outcome category exhibited the highest proportion of explicitly specified PROs (339/489, 69.3%).

**Table 3:** Number of explicitly specified patient-reported outcomes (PROs) and implicitly specified PROs used in different trial classifications (n=2390).

Classification	Explicitly specified PROs, n (%)	Implicitly specified PROs, n (%)
Primary outcome (n=641)	335 (52.3)	306 (47.7)
Secondary outcome (n=1260)	606 (48.1)	654 (51.9)
Coprimary outcome (n=489)	339 (69.3)	150 (30.7)

Tables 4-6 display the frequency of use of PRO scales for different diseases under the 3 categories. In trials using PRO instruments as coprimary outcomes, the visual analog scale (VAS) and the numeric rating scale (NRS) were the most commonly used scales for various tumors. For trials using PRO instruments as primary outcomes, the VAS was the most commonly used scale for various diseases. For trials using PRO instruments as secondary outcomes, the most commonly used scale for each disease was the European Organisation for Research and Treatment of Cancer Quality of Life Core Questionnaire-30 (EORTC QLQ-C30).

**Table 4:** Frequency of patient-reported outcome (PRO) instruments used as primary outcomes in different classifications of tumor trials by condition.

Condition	Trials (n=1280), n (%)	Instrument frequency in trials with PRO instruments as primary outcomes			
		Trials, n/N (%)	Instrument, n/N (%)	Instrument, n/N (%)	Instrument, n/N (%)
Thorax tumors	217 (16.95)	56/217 (25.81)	VAS^a^, 25/56 (44.64)	NRS^b^, 9/56 (16.07)	EORTC QLQ-LC43^c^, 4/56 (7.14)
Breast tumors	176 (13.75)	41/176 (23.3)	VAS, 14/41 (34.15)	SF-36^d^, 4/41 (9.76)	PSQI^e^, 3/41 (7.32)
Lower gastrointestinal tract tumors	173 (13.52)	43/173 (24.86)	VAS, 14/43 (32.56)	IPSS^f^, 4/43 (9.3)	LARS^g^, 4/43 (9.3)
Upper gastrointestinal tract tumors	140 (10.94)	46/140 (32.86)	VAS, 13/46 (28.26)	EORTC QLQ-C30^h^, 12/46 (26.09)	EORTC QLQ-STO22^i^, 8/46 (17.39)
Head and neck tumors	101 (7.89)	21/101 (20.79)	VAS, 3/21 (14.29)	UW-QOL^j^, 3/21 (14.29)	NRS, 2/21 (9.52)
Female reproductive organ tumors	85 (6.64)	20/85 (23.53)	VAS, 6/20 (30)	NRS, 3/20 (15)	EORTC QLQ-C30, 3/20 (15)
Hepatobiliary system tumors	67 (5.23)	14/67 (20.9)	VAS, 6/14 (42.86)	EORTC QLQ-C30, 3/14 (21.43)	QoR-40^k^, 1/14 (7.14)
Male genital organ tumors	31 (2.42)	11/31 (35.48)	VAS, 7/11 (63.64)	NRS, 3/11 (27.27)	EORTC QLQ-C30, 2/11 (18.18)
Endocrine system tumors	30 (2.34)	16/30 (53.33)	VAS, 9/16 (56.25)	IDS^l^, 2/16 (12.5)	EORTC QLQ-C30, 1/16 (6.25)
Central nervous system tumors	25 (1.95)	5/25 (20)	VAS, 2/5 (40)	QoR-40, 1/5 (20)	NRS, 1/5 (20)
Urinary tract tumors	17 (1.33)	4/17 (23.53)	VAS, 1/4 (25)	IIEF-15^m^, 1/4 (25)	QoR-15^n^, 1/4 (25)
Bone tumors	8 (0.63)	5/8 (62.5)	NRS, 2/5 (40)	VAS, 2/5 (40)	SF-36, 1/5 (20)
Hematologic malignant tumors	9 (0.7)	2/9 (22.22)	NRS, 1/2 (50)	TCMSS^o^, 1/2 (50)	N/A^p^
Neuroendocrine tumors	10 (0.78)	N/A	N/A	N/A	N/A
Skin tumors	3 (0.23)	N/A	N/A	N/A	N/A
Ophthalmic tumors	1 (0.08)	N/A	N/A	N/A	N/A
Soft tissue sarcoma	0 (0)	N/A	N/A	N/A	N/A
Other tumors	187 (14.61)	51/187 (27.27)	VAS, 18/51 (35.29)	NRS, 11/51 (21.57)	EORTC QLQ-C30, 4/51 (7.84)

^a^VAS: visual analog scale.

^b^NRS: numeric rating scale.

^c^EORTC QLQ-LC43: European Organisation for Research and Treatment of Cancer Quality of Life Questionnaire–Lung Cancer 43.

^d^SF-36: 36-item Short Form Health Survey.

^e^PSQI: Pittsburgh Sleep Quality Index.

^f^IPSS: International Prostate Symptom Score.

^g^LARS: Low Anterior Resection Syndrome.

^h^EORTC QLQ-C30: European Organisation for Research and Treatment of Cancer Quality of Life Core Questionnaire-30.

^i^EORTC QLQ-STO22: European Organisation for Research and Treatment of Cancer Quality of Life Questionnaire–Stomach 22.

^j^UW-QOL: University of Washington Quality of Life Questionnaire.

^k^QoR-40: Quality of Recovery-40.

^l^IDS: Involvement-Detachment Scale.

^m^IIEF-15: International Index of Erectile Function-15.

^n^QoR-15: Quality of Recovery-15.

^o^TCMSS: Traditional Chinese Medicine Symptom Scale.

^p^N/A: not applicable.

**Table 5:** Frequency of patient-reported outcome (PRO) instruments used as secondary outcomes in different classifications of tumor trials by condition.

Conditions	Trials (n=1280), n (%)	Instrument frequency in trials with PRO instruments as secondary outcomes			
		Trials, n/N (%)	Instrument, n/N (%)	Instrument, n/N (%)	Instrument, n/N (%)
Thorax tumors	217 (16.95)	98/217 (45.16)	EORTC QLQ-C30^a^, 33/98 (33.67)	FACT-L^b^, 18/98 (18.37)	EORTC QLQ-LC13^c^, 15/98 (15.31)
Breast tumors	176 (13.75)	67/176 (38.07)	EORTC QLQ-C30, 23/67 (34.33)	FACT-B^d^, 16/67 (23.88)	EORTC QLQ-BR23^e^, 13/67 (19.4)
Lower gastrointestinal tract tumors	173 (13.52)	94/173 (54.34)	EORTC QLQ-C30, 38/94 (40.43)	VAS^f^, 21/94 (22.34)	Wexner Scale, 11/94 (11.7)
Upper gastrointestinal tract tumors	140 (10.94)	72/140 (51.43)	EORTC QLQ-C30, 41/72 (56.94)	EORTC QLQ-OES18^g^, 15/72 (20.83)	VAS, 13/72 (18.06)
Head and neck tumors	101 (7.89)	68/101 (67.33)	EORTC QLQ-C30, 38/68 (55.88)	EORTC QLQ-H&N35^h^, 34/68 (50)	NRS^i^, 6/68 (8.82)
Female reproductive organ tumors	85 (6.64)	49/85 (57.65)	EORTC QLQ-C30, 18/49 (36.73)	VAS, 5/49 (10.2)	EORTC QLQ-CX24^j^, 5/49 (10.2)
Hepatobiliary system tumors	67 (5.23)	37/67 (55.22)	EORTC QLQ-C30, 18/37 (48.65)	EORTC QLQ-HCC18^k^, 7/37 (18.92)	VAS, 5/37 (13.51)
Male genital organ tumors	31 (2.42)	10/31 (32.26)	FACT-P^l^, 4/10 (40)	BPI-SF^m^, 3/10 (30)	FACT-G^n^, 3/10 (30)
Endocrine system tumors	30 (2.34)	7/30 (23.33)	VAS, 3/7 (42.86)	QoR-40^o^, 1/7 (14.29)	SF-36^p^, 1/7 (14.29)
Central nervous system tumors	25 (1.95)	14/25 (56)	VAS, 8/14 (57.14)	NRS, 4/14 (28.57)	QoR-15^q^, 3/14 (21.43)
Urinary tract tumors	17 (1.33)	11/17 (64.71)	EORTC QLQ-C30, 3/11 (27.27)	VAS, 3/11 (27.27)	WHOQOL-BREF^r^, 2/11 (18.18)
Neuroendocrine tumors	10 (0.78)	10/10 (100)	EORTC QLQ-C30, 6/10 (60)	EORTC QLQ-PAN26^s^, 2/10 (20)	VAS, 2/10 (20)
Hematologic malignant tumors	9 (0.7)	4/9 (44.44)	EORTC QLQ-C30, 2/4 (50)	FACIT^t^, 2/4 (50)	EQ-5D-5L, 1/4 (25)
Skin tumors	3 (0.23)	3/3 (100)	EORTC QLQ-C30, 1/3 (33.33)	VAS, 1/3 (33.33)	HF-QoL^u^, 1/3 (33.33)
Bone tumors	8 (0.63)	3/8 (37.5)	EORTC QLQ-C30, 2/3 (66.67)	BPI-SF, 1/3 (33.33)	N/A^v^
Ophthalmic tumors	1 (0.08)	1/1 (100)	EORTC QLQ-C30, 1/1 (100)	EORTC QLQ-OPT30^w^, 1/1 (100)	N/A
Soft tissue sarcoma	0 (0)	N/A	N/A	N/A	N/A
Other tumors	187 (14.61)	58/187 (31.02)	VAS, 12/58 (20.69)	EORTC QLQ-C30, 12/58 (20.69)	NRS, 9/58 (15.52)

^a^EORTC QLQ-C30: European Organisation for Research and Treatment of Cancer Quality of Life Core Questionnaire-30.

^b^FACT-L: Functional Assessment of Cancer Therapy–Lung.

^c^EORTC QLQ-LC13: European Organisation for Research and Treatment of Cancer Quality of Life Questionnaire–Lung Cancer 13.

^d^FACT-B: Functional Assessment of Cancer Therapy–Breast.

^e^EORTC QLQ-BR23: European Organisation for Research and Treatment of Cancer Quality of Life Questionnaire–Breast Cancer 23.

^f^VAS: visual analog scale.

^g^EORTC QLQ-OES18: European Organisation for Research and Treatment of Cancer Quality of Life Questionnaire–Oesophageal Cancer 18.

^h^EORTC QLQ-H&N35: European Organisation for Research and Treatment of Cancer Quality of Life Questionnaire–Head and Neck Cancer 35.

^i^NRS: numeric rating scale.

^j^EORTC QLQ-CX24: European Organisation for Research and Treatment of Cancer Quality of Life Questionnaire–Cervical Cancer 24.

^k^EORTC QLQ-HCC18: European Organisation for Research and Treatment of Cancer Quality of Life Questionnaire–Hepatocellular Carcinoma 18.

^l^FACT-P: Functional Assessment of Cancer Therapy–Prostate.

^m^BPI-SF: Brief Pain Inventory–Short Form.

^n^FACT-G: Functional Assessment of Cancer Therapy–General.

^o^QoR-40: Quality of Recovery-40.

^p^SF-36: 36-item Short Form Health Survey.

^q^QoR-15: Quality of Recovery-15.

^r^WHOQOL-BREF: World Health Organization Quality of Life Brief Version.

^s^EORTC QLQ-PAN26: European Organisation for Research and Treatment of Cancer Quality of Life Questionnaire–Pancreatic Cancer 26.

^t^FACIT: Functional Assessment of Chronic Illness Therapy.

^u^HF-QOL: Hand-Foot Skin Reaction and Quality of Life.

^v^N/A: not applicable.

^w^EORTC QLQ-OPT30: European Organisation for Research and Treatment of Cancer Quality of Life Questionnaire–Ophthalmic Cancer 30.

**Table 6:** Frequency of patient-reported outcome (PRO) instruments used as coprimary outcomes in different classifications of tumor trials by condition.

Conditions	Trials (n=1280), n (%)	Instrument frequency in trials with PRO instruments as coprimary outcomes			
		Trials, n/N (%)	Instrument, n/N (%)	Instrument, n/N (%)	Instrument, n/N (%)
Thorax tumors	217 (16.95)	63/217 (29.03)	VAS^a^, 22/63 (34.92)	NRS^b^, 17/63 (26.98)	EORTC QLQ-C30^c^, 16/63 (25.4)
Breast tumors	176 (13.75)	68/176 (38.64)	VAS, 22/68 (32.35)	NRS, 13/68 (19.12)	QoR-15^d^, 9/68 (13.24)
Lower gastrointestinal tract tumors	173 (13.52)	36/173 (20.81)	VAS, 13/36 (36.11)	EORTC QLQ-C30, 7/36 (19.44)	QoR-15, 6/36 (16.67)
Upper gastrointestinal tract tumors	140 (10.94)	22/140 (15.71)	VAS, 7/22 (31.82)	NRS, 7/22 (31.82)	EORTC QLQ-C30, 3/22 (13.64)
Head and neck tumors	101 (7.89)	12/101 (11.88)	NRS, 4/12 (33.33)	EORTC QLQ-C30, 3/12 (25)	TNSS^e^, 2/12 (16.67)
Female reproductive organ tumors	85 (6.64)	16/85 (18.82)	VAS, 6/16 (37.5)	BCS^f^, 2/16 (12.5)	NRS, 2/16 (12.5)
Hepatobiliary system tumors	67 (5.23)	16/67 (23.88)	NRS, 7/16 (43.75)	EORTC QLQ-C30, 2/16 (12.5)	PSQI^g^, 2/16 (12.5)
Male genital organ tumors	31 (2.42)	10/31 (32.26)	VAS, 3/10 (30)	ICIQ-SF^h^, 3/10 (30)	FACT-P^i^, 2/10 (20)
Endocrine system tumors	30 (2.34)	7/30 (23.33)	QoR-15, 3/7 (42.86)	VAS, 3/7 (42.86)	HADS^j^, 1/7 (14.29)
Central nervous system tumors	25 (1.95)	6/25 (24)	VAS, 5/6 (83.33)	EORTC IADL-BN32^k^, 1/6 (16.67)	N/A^l^
Urinary tract tumors	17 (1.33)	2/17 (11.76)	NRS, 1/2 (50)	QoR-15, 1/2 (50)	VAS, 1/2 (50)
Hematologic malignant tumors	9 (0.7)	3/9 (33.33)	SAS^m^, 1/3 (33.33)	SDS^n^, 1/3 (33.33)	NRS, 1/3 (33.33)
Bone tumors	8 (0.63)	N/A	N/A	N/A	N/A
Neuroendocrine tumors	10 (0.78)	N/A	N/A	N/A	N/A
Skin tumors	3 (0.23)	N/A	N/A	N/A	N/A
Ophthalmic tumors	1 (0.08)	N/A	N/A	N/A	N/A
Soft tissue sarcoma	0 (0)	N/A	N/A	N/A	N/A
Other tumors	187 (14.61)	78/187 (41.71)	NRS, 24/78 (30.77)	VAS, 14/78 (17.95)	PSQI, 12/78 (15.38)

^a^VAS: visual analog scale.

^b^NRS: numeric rating scale.

^c^EORTC QLQ-C30: European Organisation for Research and Treatment of Cancer Quality of Life Core Questionnaire-30.

^d^QoR-15: Quality of Recovery-15.

^e^TNSS: Total Nasal Symptom Score.

^f^BCS: Bruggemann Comfort Scale.

^g^PSQI: Pittsburgh Sleep Quality Index.

^h^ICIQ-SF: International Consultation on Incontinence Questionnaire–Short Form.

^i^FACT-P: Functional Assessment of Cancer Therapy–Prostate.

^j^HADS: Hospital Anxiety and Depression Scale.

^k^EORTC IADL-BN32: European Organisation for Research and Treatment of Cancer Instrumental Activities of Daily Living in Patients With Brain Tumors-32.

^l^N/A: not applicable.

^m^SAS: Self-Rating Anxiety Scale.

^n^SDS: Self-Rating Depression Scale.

To analyze the overall application of scales in explicitly specified PROs by condition, we examined the specific PRO instruments used in trials that explicitly mentioned the PRO instruments as primary or secondary outcomes (Table 7). Of the 1280 trials, 321 (25.08%) used the EORTC QLQ-C30 (Multimedia Appendix 3), which was the most commonly used PRO scale. Of note, the EORTC QLQ-C30 was the most commonly used scale in trials concerning lower gastrointestinal tract, upper gastrointestinal tract, head and neck, female reproductive organ, hepatobiliary system, bone, neuroendocrine, skin, and ophthalmic tumors as well as hematologic malignancies. In addition, the VAS was used in 24.77% (317/1280) of the trials (Multimedia Appendix 3), predominating in trials involving thorax, breast, male genital organ, endocrine system, central nervous system, and urinary tract tumors. The NRS was also frequently used (169/1280, 13.2%) in cancer trials. More targeted scales have been used for different tumor diseases; for example, the European Organisation for Research and Treatment of Cancer Quality of Life Questionnaire (EORTC QLQ)–Head and Neck Cancer 35 (36/101, 35.6%) was more common in head and neck tumor trials, the EORTC QLQ–Oesophageal Cancer 18 (15/140, 10.7%) and the EORTC QLQ–Stomach 22 (14/140, 10%) were frequently observed in upper gastrointestinal cancer trials, the EORTC QLQ–Colorectal Cancer 29 (14/173, 8.1%) scale was prevalent in lower gastrointestinal cancer trials, the EORTC QLQ–Hepatocellular Carcinoma 18 (8/67, 12%) was frequently found in hepatobiliary system tumor trials, the Functional Assessment of Cancer Therapy (FACT)–Lung (21/217, 9.7%) and the EORTC QLQ–Lung Cancer 13 (19/217, 8.8%) commonly featured in thorax tumor trials, the FACT–Breast (29/176, 16.5%) and the EORTC QLQ–Breast Cancer 23 (16/176, 9.1%) were frequently seen in breast cancer trials, the EORTC QLQ–Ovarian Cancer 28 (6/85, 7%) was a typical scale used in female reproductive organ tumor trials, the FACT–Prostate (7/31, 23%) was often used in male genital organ tumor trials, and the FACT–Anemia (1/9, 11%) and the FACT–Lymphoma (1/9, 11%) were common choices in hematologic malignant tumor trials.

**Table 7:** Frequency of use of patient-reported outcome (PRO) instruments by condition.

Conditions	Trials (n=1280), n (%)	Instrument, n/N (%)	Instrument, n/N (%)	Instrument, n/N (%)	Instrument, n/N (%)	Instrument, n/N (%)
Thorax tumors	217 (16.95)	VAS^a^, 57/217 (26.27)	EORTC QLQ-C30^b^, 53/217 (24.42)	NRS^c^, 32/217 (14.75)	FACT-L^d^, 21/217 (9.68)	EORTC QLQ-LC13^e^, 19/217 (8.76)
Breast tumors	176 (13.75)	VAS, 43/176 (24.43)	FACT-B^f^, 29/176 (16.48)	EORTC QLQ-C30, 26/176 (14.77)	EORTC QLQ-BR23^g^, 16/176 (9.09)	NRS, 15/176 (8.52)
Lower gastrointestinal tract tumors	173 (13.52)	EORTC QLQ-C30, 49/173 (28.32)	VAS, 48/173 (27.75)	NRS, 15/173 (8.67)	EORTC QLQ-CR29^h^, 14/173 (8.09)	Wexner Scale, 14/173 (8.09)
Upper gastrointestinal tract tumors	140 (10.94)	EORTC QLQ-C30, 56/140 (40)	VAS, 33/140 (23.57)	NRS, 17/140 (12.14)	EORTC QLQ-OES18^i^, 15/140 (10.71)	EORTC QLQ-STO22^j^, 14/140 (10)
Head and neck tumors	101 (7.89)	EORTC QLQ-C30, 41/101 (40.59)	EORTC QLQ-H&N35^k^, 36/101 (35.64)	NRS, 12/101 (11.88)	VAS, 11/101 (10.89)	PG-SGA^l^, 8/101 (7.92)
Female reproductive organ tumors	85 (6.64)	EORTC QLQ-C30, 21/85 (24.71)	VAS, 17/85 (20)	NRS, 6/85 (7.06)	SDS^m^, 6/85 (7.06)	EORTC QLQ-OV28^n^, 6/85 (7.06)
Hepatobiliary system tumors	67 (5.23)	EORTC QLQ-C30, 23/67 (34.33)	VAS, 13/67 (19.4)	NRS, 9/67 (13.43)	EORTC QLQ-HCC18^o^, 8/67 (11.94)	TCMSS^p^, 5/67 (7.46)
Male genital organ tumors	31 (2.42)	VAS, 10/31 (32.26)	FACT-P^q^, 7/31 (22.58)	BPI^r^, 4/31 (12.9)	IPSS^s^, 4/31 (12.9)	NRS, 4/31 (12.9)
Endocrine system tumors	30 (2.34)	VAS, 15/30 (50)	QoR-15^t^, 4/30 (13.33)	EORTC QLQ-C30, 2/30 (6.67)	NRS, 2/30 (6.67)	QoR-40^u^, 2/30 (6.67)
Central nervous system tumors	25 (1.95)	VAS, 15/25 (60)	NRS, 4/25 (16)	QoR-15, 3/25 (12)	QoR-40, 2/25 (8)	PCSQ^v^, 2/25 (8)
Urinary tract tumors	17 (1.33)	VAS, 5/17 (29.41)	EORTC QLQ-C30, 3/17 (17.65)	NRS, 2/17 (11.76)	QoR-15, 2/17 (11.76)	WHOQOL-BREF^w^, 2/17 (11.76)
Hematologic malignant tumors	9 (0.7)	EORTC QLQ-C30, 2/9 (22.22)	NRS, 2/9 (22.22)	EQ-5D, 1/9 (11.11)	FACT-An^x^, 1/9 (11.11)	FACT-Lym^y^, 1/9 (11.11)
Bone tumors	8 (0.63)	EORTC QLQ-C30, 2/8 (25)	VAS, 2/8 (25)	NRS, 2/8 (25)	BPI, 1/8 (12.5)	SF-36^z^, 1/8 (12.5)
Neuroendocrine tumors	10 (0.78)	EORTC QLQ-C30, 6/10 (60)	EORTC QLQ-PAN26^aa^, 2/10 (20)	VAS, 2/10 (20)	N/A^ab^	N/A
Skin tumors	3 (0.23)	EORTC QLQ-C30, 1/3 (33.33)	VAS, 1/3 (33.33)	HF-QoL^ac^, 1/3 (33.33)	N/A	N/A
Ophthalmic tumors	1 (0.08)	EORTC QLQ-C30, 1/1 (100)	EORTC QLQ-OPT30^ad^, 1/1 (100)	N/A	N/A	N/A
Soft tissue sarcoma	0 (0)	N/A	N/A	N/A	N/A	N/A
Other tumors	187 (14.61)	VAS, 44/187 (23.53)	NRS, 44/187 (23.53)	EORTC QLQ-C30, 33/187 (17.65)	PSQI^ae^, 15/187 (8.02)	BFI^af^, 11/187 (5.88)

^a^VAS: visual analog scale.

^b^EORTC QLQ-C30: European Organisation for Research and Treatment of Cancer Quality of Life Core Questionnaire-30.

^c^NRS: numeric rating scale.

^d^FACT-L: Functional Assessment of Cancer Therapy–Lung.

^e^EORTC QLQ-LC13: European Organisation for Research and Treatment of Cancer Quality of Life Questionnaire–Lung Cancer 13.

^f^FACT-B: Functional Assessment of Cancer Therapy–Breast.

^g^EORTC QLQ-BR23: European Organisation for Research and Treatment of Cancer Quality of Life Questionnaire–Breast Cancer 23.

^h^EORTC QLQ-CR29: European Organisation for Research and Treatment of Cancer Quality of Life Questionnaire–Colorectal Cancer 29.

^i^EORTC QLQ-OES18: European Organisation for Research and Treatment of Cancer Quality of Life Questionnaire–Oesophageal Cancer 18.

^j^EORTC QLQ-STO22: European Organisation for Research and Treatment of Cancer Quality of Life Questionnaire–Stomach 22.

^k^EORTC QLQ-H&N35: European Organisation for Research and Treatment of Cancer Quality of Life Questionnaire–Head and Neck Cancer 35.

^l^PG-SGA: Patient-Generated Subjective Global Assessment.

^m^SDS: Self-Rating Depression Scale.

^n^EORTC QLQ-OV28: European Organisation for Research and Treatment of Cancer Quality of Life Questionnaire–Ovarian Cancer 28.

^o^EORTC QLQ-HCC18: European Organisation for Research and Treatment of Cancer Quality of Life Questionnaire–Hepatocellular Carcinoma 18.

^p^TCMSS: Traditional Chinese Medicine Symptom Scale.

^q^FACT-P: Functional Assessment of Cancer Therapy–Prostate.

^r^BPI: Brief Pain Inventory.

^s^IPSS: International Prostate Symptom Score.

^t^QoR-15: Quality of Recovery-15.

^u^QoR-40: Quality of Recovery-40.

^v^PCSQ: Preparedness for Colorectal Cancer Surgery Questionnaire.

^w^WHOQOL-BREF: World Health Organization Quality of Life Brief Version.

^x^FACT-An: Functional Assessment of Cancer Therapy–Anemia.

^y^FACT-Lym: Functional Assessment of Cancer Therapy–Lymphoma.

^z^SF-36: 36-item Short Form Health Survey.

^aa^EORTC QLQ-PAN26: European Organisation for Research and Treatment of Cancer Quality of Life Questionnaire–Pancreatic Cancer 26.

^ab^N/A: not applicable.

^ac^HF-QoL: Hand-Foot Skin Reaction and Quality of Life.

^ad^EORTC QLQ-OPT30: European Organisation for Research and Treatment of Cancer Quality of Life Questionnaire–Ophthalmic Cancer 30.

^ae^PSQI: Pittsburgh Sleep Quality Index.

^af^BFI: Brief Fatigue Inventory.

## Discussion

### Principal Findings

This cross-sectional study depicted the general characteristics of adult tumor clinical trials incorporating PROs in China and analyzed the application of PRO instruments in randomized clinical trials of tumors to provide potential directions for future research and serve as a reference for tumor clinical practice. The findings revealed that a significant proportion, specifically 62.92% (4055/6445) of the included trials, missed the opportunity to capture patients’ subjective evaluations. Of the trials with PRO instruments as end points, 26.82% (641/2390) used PRO instruments as primary outcomes, 52.72% (1260/2390) as secondary outcomes, and 20.46% (489/2390) as coprimary outcomes. The majority of PRO trials (2144/2390, 89.71%) used 1 to 3 PRO instruments. Given that PROs can authentically represent patients’ subjective experiences and evaluations, they should receive heightened emphasis in the context of tumor clinical trials. However, in light of the small proportion of tumor-related randomized clinical trials assessing PROs, policy makers and standard-setting bodies are recommended to further promote the collection of PROs in such trials in China.

This study delved into the yearly distribution of tumor clinical trials, indicating a notable surge in the use of PRO instruments as end points between January 1, 2010, and June 30, 2022. Among the trials incorporating PROs, early phase trials constituted the largest proportion (575/2390, 24.06%), followed by phase 3 (284/2390, 11.88%) and phase 4 (269/2390, 11.26%) trials. A retrospective cross-sectional study suggested a potential correlation between the use of PROs in late-stage trials and improved drug outcomes, such as overall survival [25]. However, the omission of PROs in late-stage trial results may reduce the value of patient participation in these trials. Previous work has shown that the concern regarding funding for PRO research seems significant, and additional funding was needed—and considered important—to pay for the use of PRO instruments to collect relevant data [26]. This may also be the reason why, among the included studies, there were few PRO tumor trials funded by industry. Relevant policies could provide more financial support for PRO tumor trials. In addition, our study indicated that the application of PRO instruments was more prevalent in trials involving drug interventions. PRO instruments can serve as valuable tools for assessing patient experiences during treatment, which is an essential aspect of drug discovery [27], and their absence can result in the exclusion of critical information, such as opportunities for patient-centered support programs and insights into benefit-risk profiles [27].

In accordance with prior research [23], our study also identified regional differences in the use of PROs. Tumor trials were more prevalent in the eastern, northern, and southern regions of China—especially in Shanghai, Beijing, Guangdong, and Jiangsu—and the adoption of PRO measurements followed a similar pattern. Conversely, in other regions of China, especially in the northwestern and northeastern regions—such as Qinghai, Tibet and Heilongjiang—both the overall number of tumor clinical trials and those incorporating PRO instruments as end points were conspicuously lower. These results indicated the relationship between the volume of tumor clinical trials and the adoption of PRO tools. In addition, other factors such as economic conditions and medical resources also played an important role in this phenomenon [28]. Relevant policies can continue to encourage medical resources to be tilted toward rural and less developed areas. Remarkably, the study suggested that in resource-constrained remote regions, simplified applications of PRO instruments may be considered in tumor clinical trials. Moreover, our investigation revealed a lower prevalence of industry-funded trials in tumor clinical trials in China. This discrepancy may be attributed to previous findings that tumor trials were characterized by increased risk and costliness [29].

This study further found that thorax tumors, breast tumors, and lower gastrointestinal tract tumors were the most common conditions in trials with explicit PRO instruments. This might be related to variances in tumor incidence and different clinical concerns [30]. In the primary and coprimary outcome trial sets, a higher proportion of trials explicitly listed the PRO instruments as end points compared to those not specifying PROs, underscoring the normative inclination to formalize the acquisition and application of PRO instruments. Adherence to guidelines and standardization of PRO application is essential to maximize the application of PRO trial data, enhance their impact, and minimize research waste [31]. In particular, studies have shown that the standardized PROs were conducive to making trials or clinical treatments more scientifically rigorous and ethically sound [32-35]. Therefore, the need to standardize the application of PRO instruments remains important, with an increased emphasis on explicitly specifying PRO instruments in clinical trials.

This study analyzed the frequency of the use of PRO instruments in different classifications of trials by medical condition and found that the VAS and the NRS were the most commonly used in trials where PROs were designated as coprimary outcomes. Meanwhile, in all trials that used PRO instruments as outcomes, the VAS and the NRS were consistently prevalent. This prevalence can be attributed to the precision, simplicity, and sensitivity of VAS scores, as well as the ease of use and standardized format of the NRS for assessing subjective indicators [36-38]. In addition, almost 90% of patients with cancer would experience pain during the course of their illness [39]. The pain is both prevalent and burdensome for patients, but there is a lack of objective evaluation indices available for this purpose [40,41]. Consequently, the VAS emerged as the preferred choice for pain assessment in clinical research. Similarly, the NRS, with its user-friendly nature and standardized format, has been the preferred tool for pain assessment [36-38]. PROs continue to represent the gold standard for evaluating patients’ core pain outcomes [42-44]. In this study, among the trials that used PRO instruments as secondary outcomes, the EORTC QLQ-C30 was the most commonly used (223/606, 36.8%), which might be attributed to the significance of addressing quality-of-life concerns for patients with tumors. This study also scrutinized the prevalent PRO instruments used in various medical conditions and found that the quality-of-life scale was frequently used in clinical trials involving tumors. The high frequency of the EORTC QLQ-C30 and FACT scale groups underscored the widespread application of these instruments in assessing patients’ quality of life in cancer clinical trials in China. Specific modules in the EORTC QLQ scale system, such as the EORTC QLQ–Breast Cancer 23, the EORTC QLQ–Lung Cancer 13, and the EORTC QLQ–Colorectal Cancer 29, have been widely used in various cancer diseases [45,46]. Similarly, specific modules in the FACT scales, such as FACT–Lung (lung cancer), FACT–Breast (breast cancer), and FACT–Prostate (prostate cancer), have exhibited a high rate of use in cancer clinical trials in China. The extensive use of various PRO scales indicates a growing awareness and acceptance of PRO instruments, which, in turn, encourages the development of more effective and reliable PRO instruments. PRO instruments can be divided into universal and disease-specific PRO instruments. Considering the heterogeneity of symptom types in patients with tumors, symptom assessment should be performed for specific diseases [47]. However, in different tumor trials, the explicitly specified PRO instruments were primarily quality-of-life scales, the VAS, and the NRS, suggesting a need for the application of disease-specific PRO scales for different tumor types in clinical trials. It is suggested that according to the heterogeneity of diseases, experts from different fields should be brought together to develop or improve the disease-specific scale through participatory and consensus approaches under the guidance of relevant guidelines [33,47,48]. Acceptance of the scale by a wide range of stakeholders would be beneficial to improve the quality and specificity of the scale [48]. Training of clinicians and researchers on disease-specific scales is recommended. In addition, regarding the implementation of PRO measurement, it can be attempted as part of routine clinical care delivery for corresponding diseases, as well as continuous quality improvement as a clinical care priority [48].

This study undertook an in-depth analysis of the fundamental aspects of tumor clinical trials encompassing PROs in China, involving categorizing tumors and assessing the application of specific PRO tools for each tumor type. The findings underscore the critical importance of integrating PRO measures into tumor clinical trials in China and the need to standardize the use of PRO instruments within these trials. In recent years, the Chinese government has attached great importance to the application of PRO instruments in clinical trials. To encourage the patient-centered concept of new drug development and make reasonable use of PRO instruments, the National Medical Products Administration formulated the *Guiding Principles for the Application of Patient Reported Outcomes in Drug Clinical Research and Development* in 2022. To further promote these guiding principles, the relevant departments can educate researchers about the importance of regulating the application of PRO instruments, provide an interpretation of these principles to researchers, and advise them to follow the guidelines. We encourage researchers to communicate relevant information to regulators in a timely manner to conduct higher-quality clinical trials, such as the background of the study, the type of study, and the scale used. Policy makers should further formulate and implement pertinent policies, and PRO application platforms need to be developed and promoted to accelerate rational use of PROs in tumor clinical trials. It is recommended to define or form an institution or department to coordinate and standardize the use of PROs in clinical trials [49]. The institution or department can provide researchers with some support, such as methodological guidance for PRO applications, interpretation of relevant guidelines, and guidance on internet technologies. Efforts should also be made to encourage communication and collaboration among policy makers, researchers, and medical institutions to promote the high-quality application of PROs in clinical trials. Furthermore, it is crucial to train clinicians in how to use PRO instruments in clinical practice. Ideally, this training can be part of standard medical education programs in the future. The most successful and effective way of training involved real patient cases and problem-based learning using audio and video clips, which could enable clinicians to know how to use PRO instruments and refer to the PRO data [50]. Researchers are encouraged to follow relevant guidelines and principles and actively engage in conducting high-quality tumor clinical trials to improve well-established PRO protocols and enrich the array of available PRO instruments, thereby advancing personalized population health. In addition, it is suggested to encourage and provide relevant support to patients who have difficulties in completing the PRO reports [51].

### Limitations

It is important to acknowledge several limitations to this study. First, we excluded trials lacking detailed end point information, which may have introduced bias into the results. Second, the inclusion of trials that have not yet commenced participant recruitment, although necessary for our investigation, may have inflated the reported outcomes. Finally, the exclusion of trials involving children due to their limited expressive ability and the potential influence of parental reporting on outcomes may have introduced bias in the findings.

### Conclusions

In China, the incorporation of PROs has demonstrated an upward trajectory in adult randomized clinical trials of tumors in recent years. Nonetheless, the infrequent measurement of the patient’s voice remains noteworthy. This study highlights the need for a more comprehensive evaluation of patients’ experiences in adult tumor clinical trials in China. The incorporation of patients’ subjective feelings in the context of tumor diseases is necessary. Disease-specific PRO instruments should be widely used in different categories of tumor disease. Pertinent policies should be formulated and implemented, and PRO application platforms need to be developed and promoted as well. In addition, researchers should actively engage in conducting high-quality tumor clinical trials. There is room for improvement in the standardization of PROs in China.

## Appendix

Multimedia Appendix 1 Classification of specific diseases.

Multimedia Appendix 2 The number of trials with patient-reported outcomes in each province of China.

Multimedia Appendix 3 Patient-reported outcome tests used most frequently.

## Abbreviations

CONSORT-PRO: Consolidated Standards of Reporting Trials–Patient-Reported Outcome
EORTC QLQ: European Organisation for Research and Treatment of Cancer Quality of Life Questionnaire
EORTC QLQ-C30: European Organisation for Research and Treatment of Cancer Quality of Life Core Questionnaire-30
FACT: Functional Assessment of Cancer Therapy
PRO: patient-reported outcome
PRO-CTCAE: Patient-Reported Outcomes version of the Common Terminology Criteria for Adverse Events
SISAQOL: Setting International Standards in Analysing Patient-Reported Outcomes and Quality of Life Endpoints
SPIRIT-PRO: Standard Protocol Items: Recommendations for Interventional Trials–Patient-Reported Outcome
STROBE: Strengthening the Reporting of Observational Studies in Epidemiology

## Data Availability

The data sets generated and analyzed during this study are available from the corresponding author on reasonable request.
