# Supplementary material for: Application of Patient-Reported Outcome Measurements in Adult Tumor Clinical Trials in China: Cross-Sectional Study
Source: J Med Internet Res. 2024 May 8;26:e45719. doi: 10.2196/45719 (PMC11112474; doi:10.2196/45719)
Supplement: Multimedia Appendix 1 [file jmir_v26i1e45719_app1.pdf]

Multimedia Appendix 1. Classification of specific diseases.

	Classification	Specific Diseases				
1	Head and Neck	Staging Head and Neck Cancers	Cervical Lymph Nodes and Unknown Primary Tumors of the Head and Neck	Lip and Oral Cavity	Major Salivary Glands	Nasopharynx
		HPV-Mediated(p16+) Oropharyngeal Cancer	Oropharynx(p16-) and Hypopharynx	Nasal Cavity and Paranasal Sinuses	Larynx	Mucosal Melanoma of the Head and Neck
		Cutaneous Squamous Cell Carcinoma of the Head and Neck				
2	Upper Gastrointestinal Tract	Esophagus and Esophagogastric Junction	Stomach	Small Intestine		
3	Lower Gastrointestinal Tract	Appendix-Carcinoma	Colon and Rectum	Anus		
4	Hepatobiliary System	Liver	Intrahepatic Bile Ducts	Gallbladder	Perihilar Bile Ducts	Distal Bile Duct
		Ampulla of Vater	Exocrine Pancreas			
5	Neuroendocrine Tumors	Neuroendocrine Tumors of the Stomach	Neuroendocrine Tumors of the Duodenum and Ampulla of Vater	Neuroendocrine Tumors of the Jejunum and ileum	Neuroendocrine Tumors of the Appendix	Neuroendocrine Tumors of the Colon and Rectum
		Neuroendocrine Tumors of the Pancreas				
6	Thorax	Thymus	Lung	Malignant Pleural Mesothelioma		
7	Bone	Bone				
8	Soft Tissue Sarcoma	Introduction to Soft Tissue Sarcoma	Soft Tissue Sarcoma of the Head and Neck	Soft Tissue Sarcoma of the Trunk and Extremities	Soft Tissue Sarcoma of the Abdomen and Thoracic Visceral Organs	Gastrointestinal Stromal Tumor
		Soft Tissue Sarcoma of the Retroperitoneum	Soft Tissue Sarcoma-Unusual Histologies and Sites			
9	Skin	Merkel Cell Carcinoma	Melanoma of the Skin			

10	Breast	Breast.				
11	Female Reproductive Organs	Introduction to Female Reproductive Organs	Vulva	Vagina	Cervix Uteri	Corpus Uteri-Carcinoma and Carcinosarcoma
		Corpus Uteri-Sarcoma	Ovary, Fallopian Tube. and Primary Peritoneal Carcinoma	Gestational Trophoblastic		
12	Male Genital Organs	Penis	Prostate	Testis		
13	Urinary Tract	Kidney	Renal Pelvis and Ureter	Urinary Bladder	Urethra	
14	Ophthalmic Sites	Eyelid Carcinoma	Conjunctival Carcinoma	Conjunctival Melanoma	Uveal Melanoma	Retinoblastoma
		Lacrimal Gland Carcinoma	Orbital Sarcoma	Ocular Adnexal Lymphoma		
15	Central Nervous System	Brain and Spinal Cord				
16	Endocrine System	Thyroid-Differentiated and Anaplastic Carcinoma	Thyroid-Medullary	Parathyroid	Adrenal Cortical Carcinoma	Adrenal-Neuroendocrine Tumors
17	Hematologic Malignancies	Introduction to Hematologic Malignancies	Hodgkin and Non-Hodgkin Lymphomas	Pediatric Hodgkin and Non-Hodgkin Lymphomas	Primary Cutaneous Lymphomas	Plasma Cell Myeloma and Plasma Cell Disorders
		Leukemia				
	Others					
