# Supplementary figures and images for: Application of Patient-Reported Outcome Measurements in Adult Tumor Clinical Trials in China: Cross-Sectional Study
Source: J Med Internet Res. 2024 May 8;26:e45719. doi: 10.2196/45719 (PMC11112474; doi:10.2196/45719)

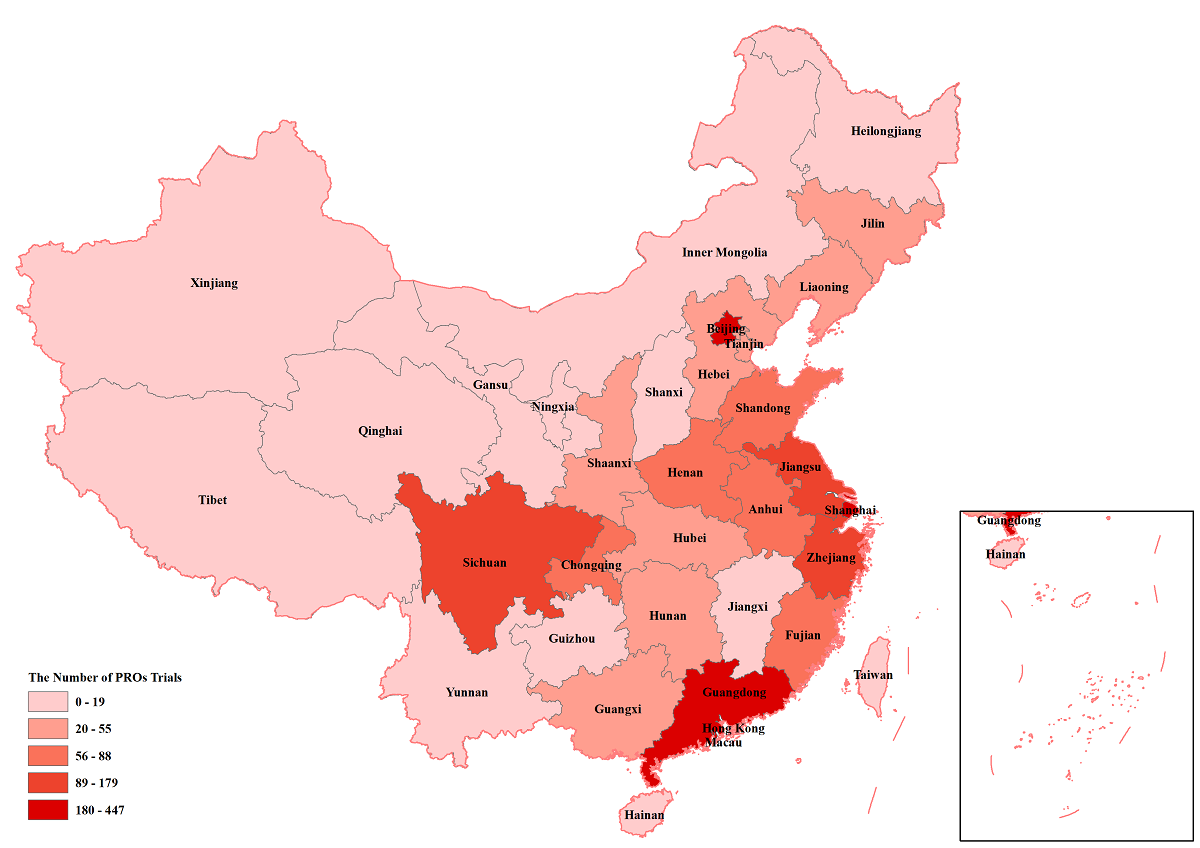

Supplement: Multimedia Appendix 2 [file jmir_v26i1e45719_app2.png]
