# Supplementary material for: Application of Patient-Reported Outcome Measurements in Adult Tumor Clinical Trials in China: Cross-Sectional Study
Source: J Med Internet Res. 2024 May 8;26:e45719. doi: 10.2196/45719 (PMC11112474; doi:10.2196/45719)
Supplement: Multimedia Appendix 3 [file jmir_v26i1e45719_app3.pdf]

Multimedia Appendix 3. Patient-reported outcome tests used most frequently.

Test	Frequency of Use, No. (%)
EORTC QLQ-C30	321 (25.1)
VAS	317 (24.8)
NRS	169 (13.2)
TCMSS	56 (4.4)
PSQI	46 (3.6)
HADS	42 (3.6)
QoR-15	42 (3.3)
SF-36	40 (3.1)
EORTC QLQ-H&N35	36 (2.8)
QoR-40	33(2.6)

Abbreviations: HADS, Hospital Anxiety and Depression Scale; NRS, Numeric Rating Scale; PSQI, Pittsburgh Sleep Quality Index; EORTC QLQ-C30, European Organization for Research and Treatment of Cancer Quality of Life Questionnaire-Core 30; EORTC QLQ-H&N35, European Organization for Research and Treatment of Cancer Quality of Life Questionnaire- Head and Neck Cancer Module; QoR-15, 15-item quality of recovery questionnaire; QoR-40, quality of recovery-40;SF-36, Short-Form 36-item Health Survey; TCMSS, Traditional Chinese Medicine Symptom Score; VAS, Visual Analog Scale.
